# Supplementary material for: Human brain prefrontal cortex proteomics identifies compromised energy metabolism and neuronal function in Schizophrenia
Source: Nat Commun. 2026 Jan 29;17:2131. doi: 10.1038/s41467-026-68950-y (PMC12957516; doi:10.1038/s41467-026-68950-y)
Supplement: Supplementary file 1 — Supplementary Information [file 41467_2026_68950_MOESM1_ESM.pdf]

Supporting information

Human brain prefrontal cortex proteomics identifies compromised energy metabolism and neuronal function in Schizophrenia

Frank Koopmans[1], Anke A. Dijkstra[2,3], Wei-Ping Li[1,4], Remco V. Klaassen[1], Yvonne Gouwenberg[1], Shuyang Yao[5], Lisa Bast[6], Matthijs Verhage[7,8], Robert Karlsson[5], Andrew J. Dwork[9], Craig A. Stockmeier[10], Jens Hjerling-Leffler[6], Patrick F. Sullivan[5,11], August B. Smit[1*]

1. Department of Molecular and Cellular Neurobiology, Center for Neurogenomics and Cognitive Research (CNCR), Vrije Universiteit (VU) Amsterdam, Amsterdam, The Netherlands.
2. Department of Pathology, Amsterdam University Medical Center (UMC), Amsterdam, The Netherlands.
3. Swammerdam Institute for Life Sciences, University of Amsterdam, Amsterdam, Netherlands.
4. Department of Complex Trait Genetics, Center for Neurogenomics and Cognitive Research (CNCR), Vrije Universiteit (VU) Amsterdam, Amsterdam, The Netherlands.
5. Department of Medical Epidemiology and Biostatistics, Karolinska Institutet, Stockholm, Sweden.
6. Department of Medical Biochemistry and Biophysics, Division of Molecular Neurobiology, Karolinska Institutet, Stockholm 17177, Sweden.
7. Department of Functional Genomics, Center for Neurogenomics and Cognitive Research (CNCR), Vrije Universiteit (VU) Amsterdam, Amsterdam, The Netherlands.
8. Department of Human Genetics, Amsterdam University Medical Center (UMC), Amsterdam, The Netherlands.
9. Department of Pathology and Cell Biology, Columbia University, New York, NY, USA.
10. Department of Psychiatry and Human Behavior, University of Mississippi Medical Center, Jackson, MS, USA.
11. Department of Genetics, University of North Carolina, Chapel Hill, NC, 27599-7264, USA.

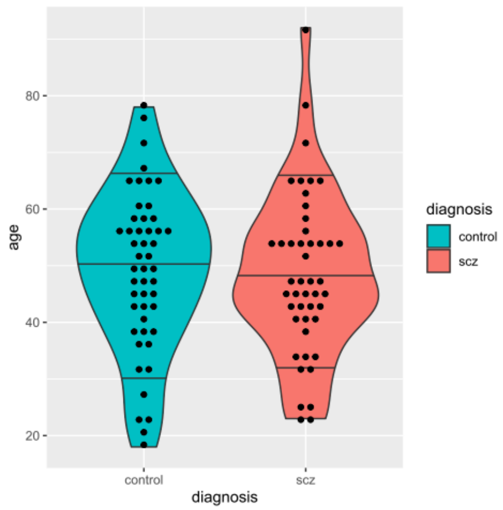

Figure S1. Age distributions of control- and patient-donors are highly similar.

The median age for *N*=49 controls (green) and *N*=47 patients (red) is 50 and 47.5 years old, respectively. Data available in Supplementary Data 1.

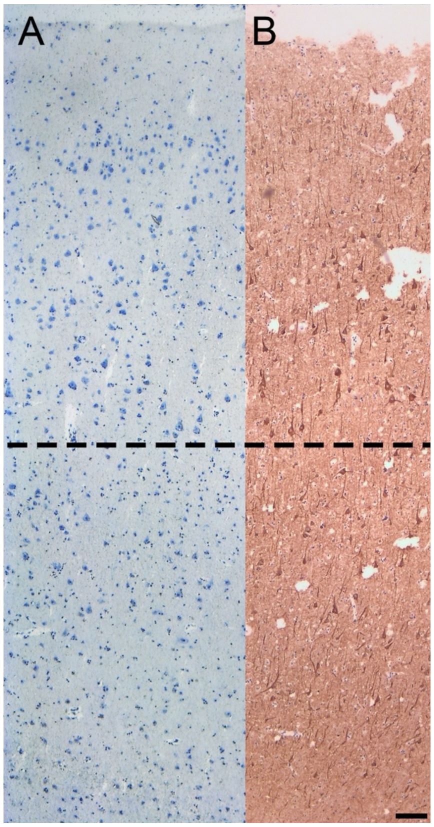

Figure S2. Cortical lamination of sections used for LCM.

Based on the Toluidine blue (**A**), Layer 4 could be determined for most cases by its granular appearance. *SMI32* (**B**) staining further confirmed the lower border of Layer 3 where large *SMI32*-positive neurons are present. The lower border of Layer 6 was determined by the Toluidine blue staining. Scale bar A,B = 100 mM.

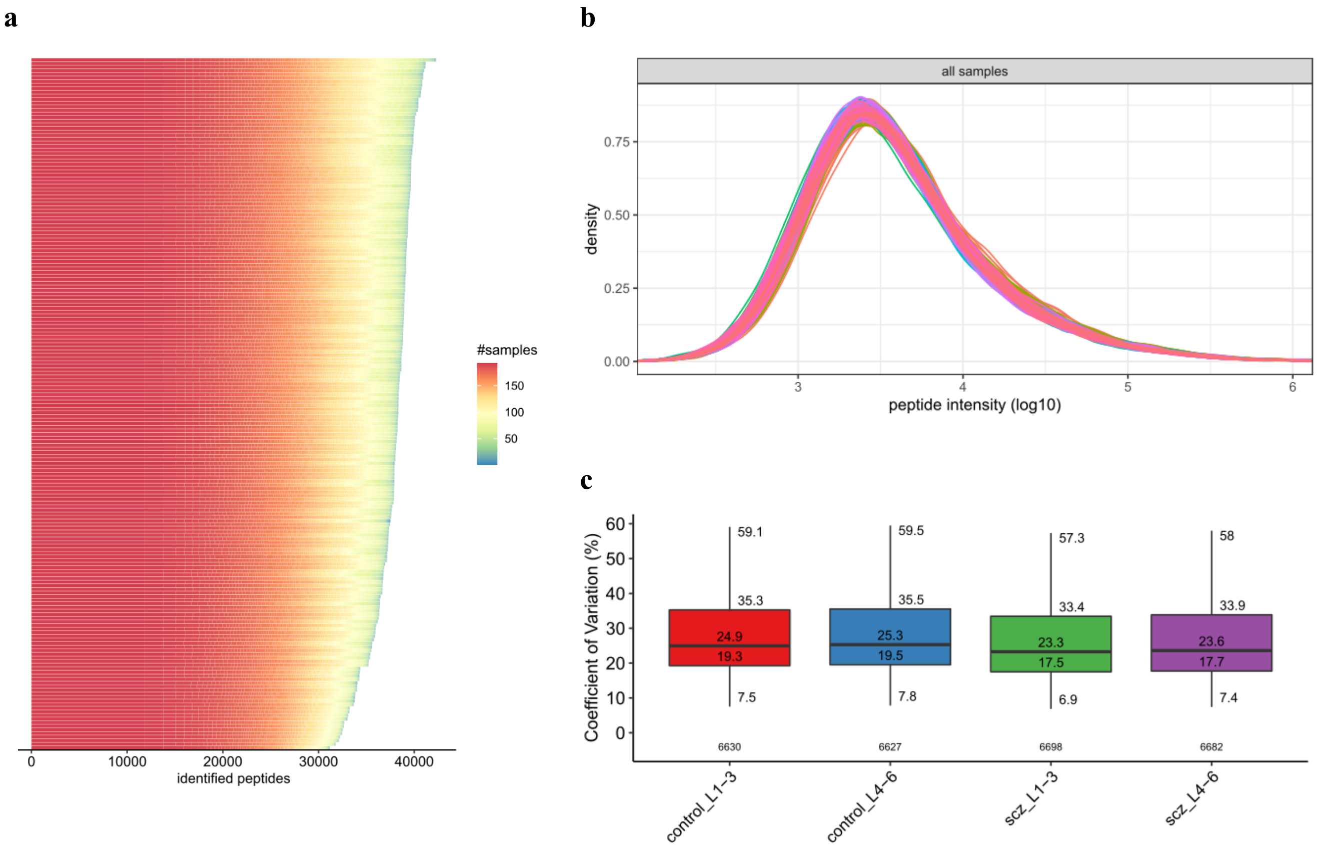

Figure S3. Quality control analyses generated by MS-DAP.

a) Identified peptide counts over all samples, color coded by the number of detections made in other samples (blue indicates peptides that are rarely detected throughout the entire dataset). **b**) Peptide intensity distributions show strong overlap for all samples, indicating that the protein sample quantities loaded into the mass-spectrometer were highly consistent. **c**) Distributions of the protein-level Coefficient of Variation (CoV) across replicates within each experimental condition. Protein counts (*N*) used for CoV computation per group are shown below each boxplot. The Layer sets, within control and schizophrenia groups, are abbreviated as L1-3 and L4-6.

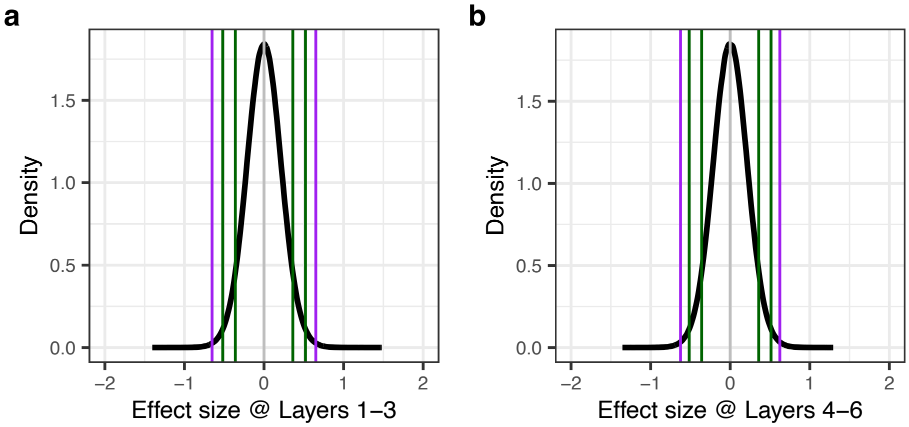

Figure S4. Bootstrapping of control versus schizophrenia regression analyses.

Sample labels (metadata) were randomly permuted and linear regression analyses (Methods) were repeated across 1000 bootstrap iterations to generate distributions of estimated protein effect sizes for the control versus schizophrenia comparison. Distributions are shown for Layers 1-3 (**a**) and Layers 4-6 (**b**). Green vertical lines indicate the 1%, 5%, 95% and 99% quantiles of the bootstrapped distributions. Purple vertical lines indicate the smallest absolute effect sizes (within up- or down-regulation) observed in the actual linear regression model for the subset of proteins significant at FDR adjusted p-value 0.05 (Fig. 1b-c, Supplementary Data 2), i.e. the primary results reported in the manuscript.

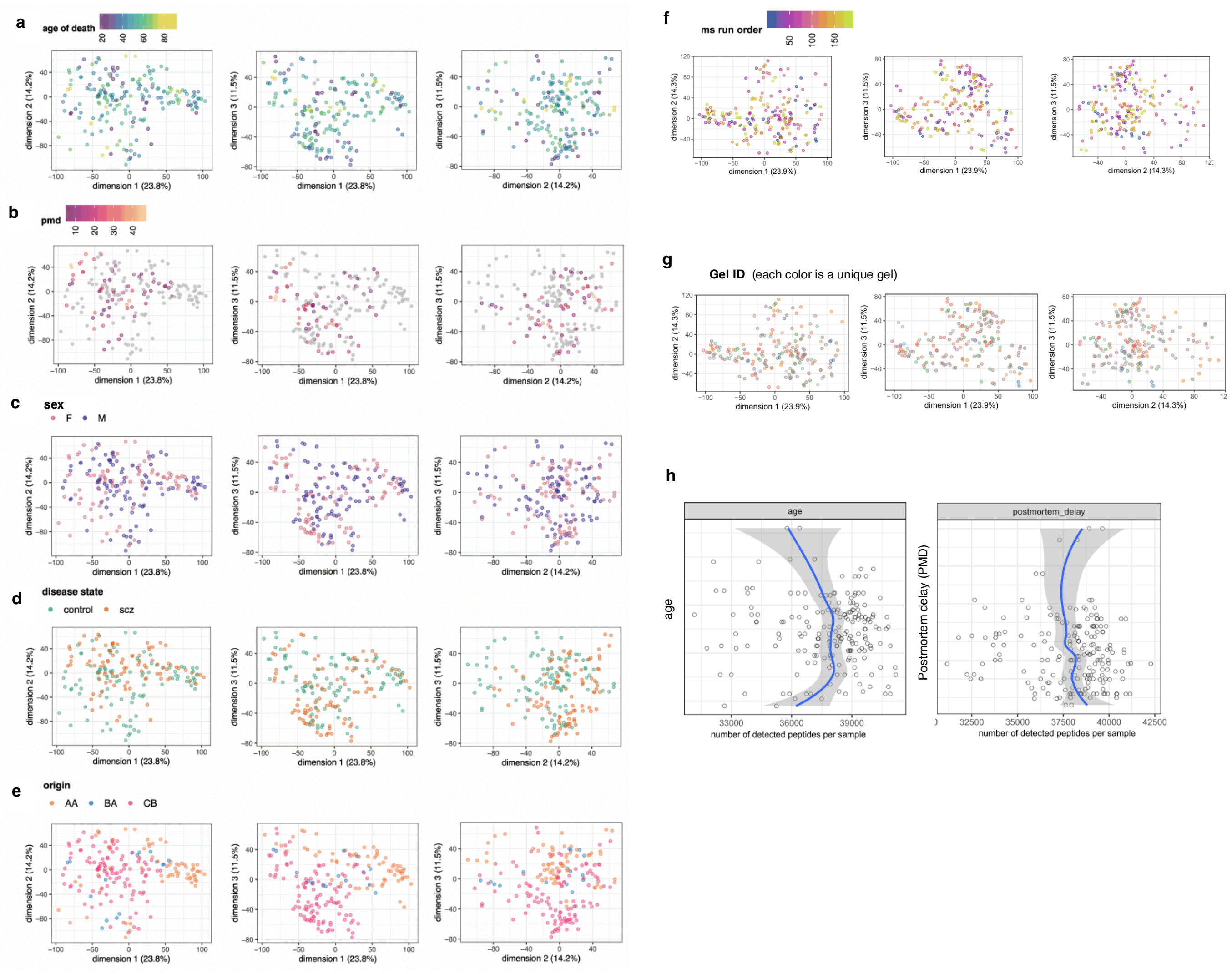

Figure S5. Quality control analyses for potential confounders generated by MS-DAP.

a-g) PCA plots of various covariates, including potential confounders of the analysis. Each dot represents an individual sample. Samples cluster only according to their diagnosis (**d**) and origin (**e**, source where the samples are obtained from; Stockmeier (CB), Netherlands Brain Bank (BA), Dwork (AA)). Postmortem delay (PMD) information was only available for a subset of samples (grey dots). **h**) Detection of the number of peptides is not dependent on age (of death) or postmortem delay (PMD). MS: mass spectrometer.

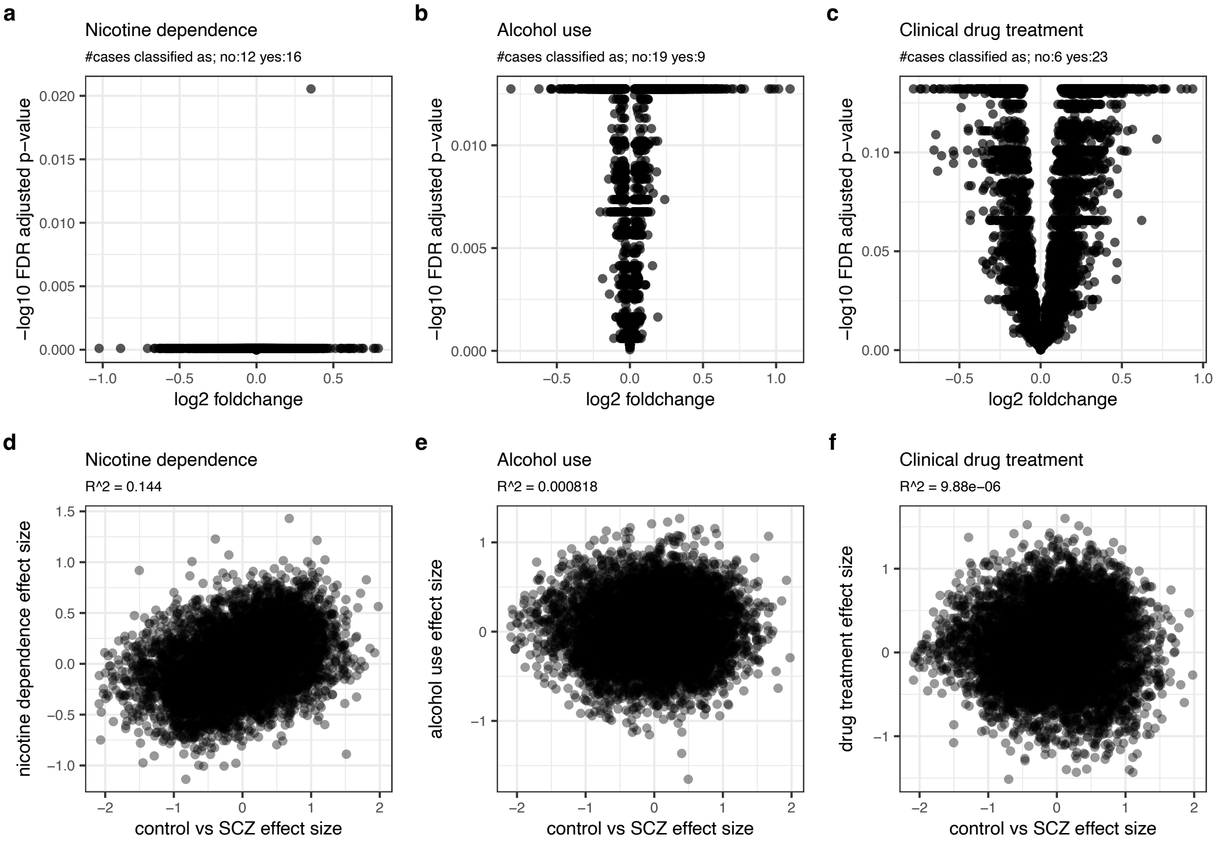

Figure S6. Statistical analysis of potential confounding effects.

a) Differential expression analysis was performed using limma linear regression models, with DEqMS correction for peptide counts per protein, to assess the impact of nicotine dependence (yes or no) on protein abundance for samples within the Stockmeier cohort (*N*=56). Both Layer sets were included in the analyses, accounting for repeated measurements from the same individual using a block design (Methods). Protein log2 foldchanges are shown on x-axis, FDR adjusted p-values are shown on the y-axis. **b-c**) analogous analyses for alcohol use (yes or no) and drug treatment (yes or no), respectively. Note that after p-value adjustment for multiple testing, p-values are far from significant as evidence from the y-axis of panels **a-c**. Sample metadata are described in Supplementary Data 1. **d-f**) Correlation plots showing effect sizes from the analyses from panels **a-c**, against effect sizes from control versus SCZ analyses (also across both Layer sets), further supporting the lack of confounding by these variables. Source data are provided as a Source Data file.

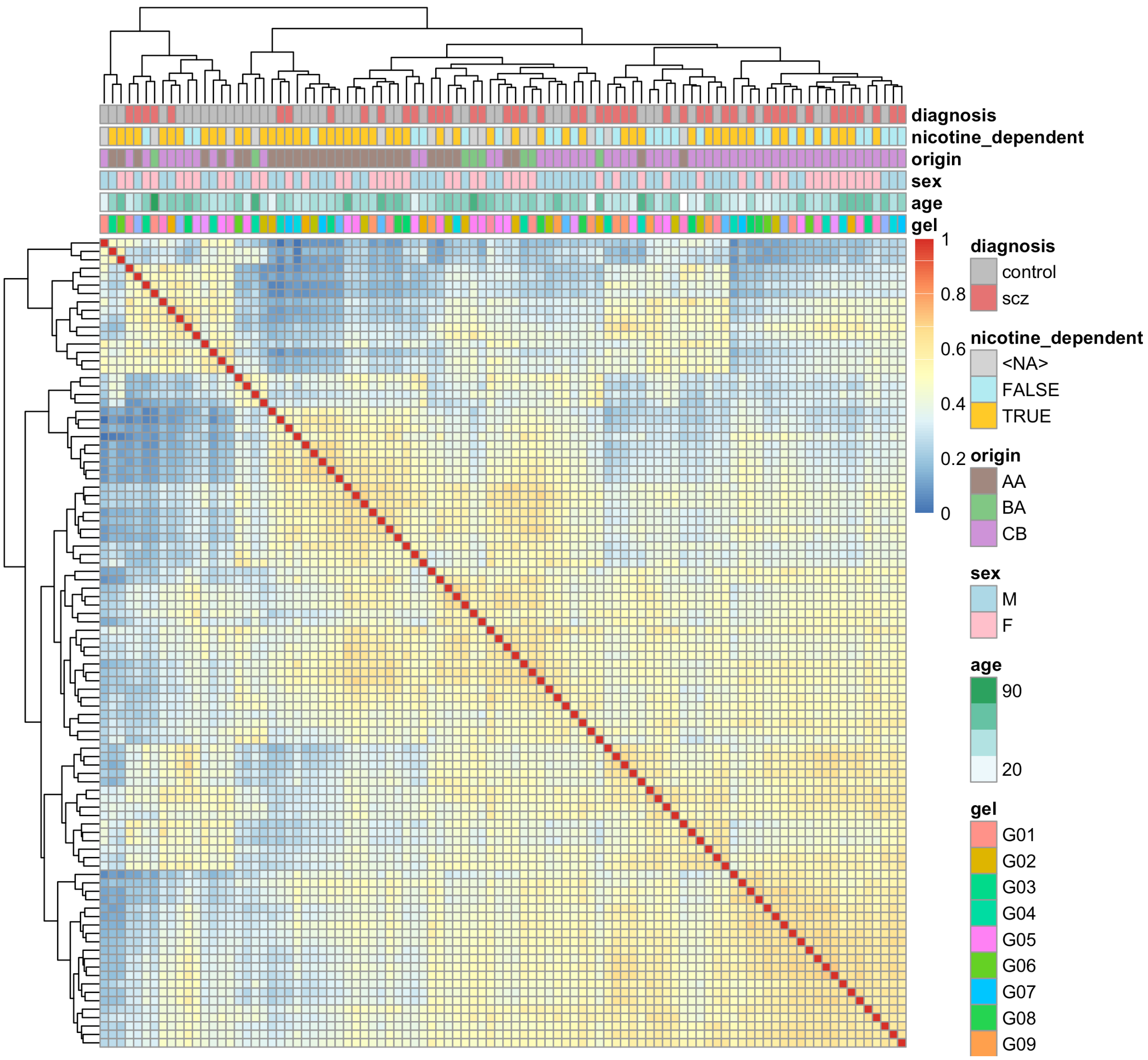

Figure S7. Unsupervised clustering of Layer 1-3 samples across all individuals.

The protein abundance (log2 intensity) matrix for all samples from Layers 1-3 was used to compute the sample similarity (1 - Euclidean distance) matrix. This sample*sample similarity matrix was used to perform unsupervised clustering analysis of individuals using the pheatmap R package with default options. Colors indicate sample similarity scores between pairs of samples. Annotated sample metadata from Supplementary Data 1 is shown as color-coded bars on top; diagnosis (control or schizophrenia/SCZ), nicotine dependence (TRUE, FALSE, Not Available), age, cohort (the source where samples were collected), sex (Male, Female), age, gel (included to check for a potential technical batch in sample preparation). Only 1 strong cluster is observed (top-left), which could not be related to any of the annotated metadata and is a mix of diagnoses; we observe no apparent SCZ patient subclusters. Source data are provided as a Source Data file.

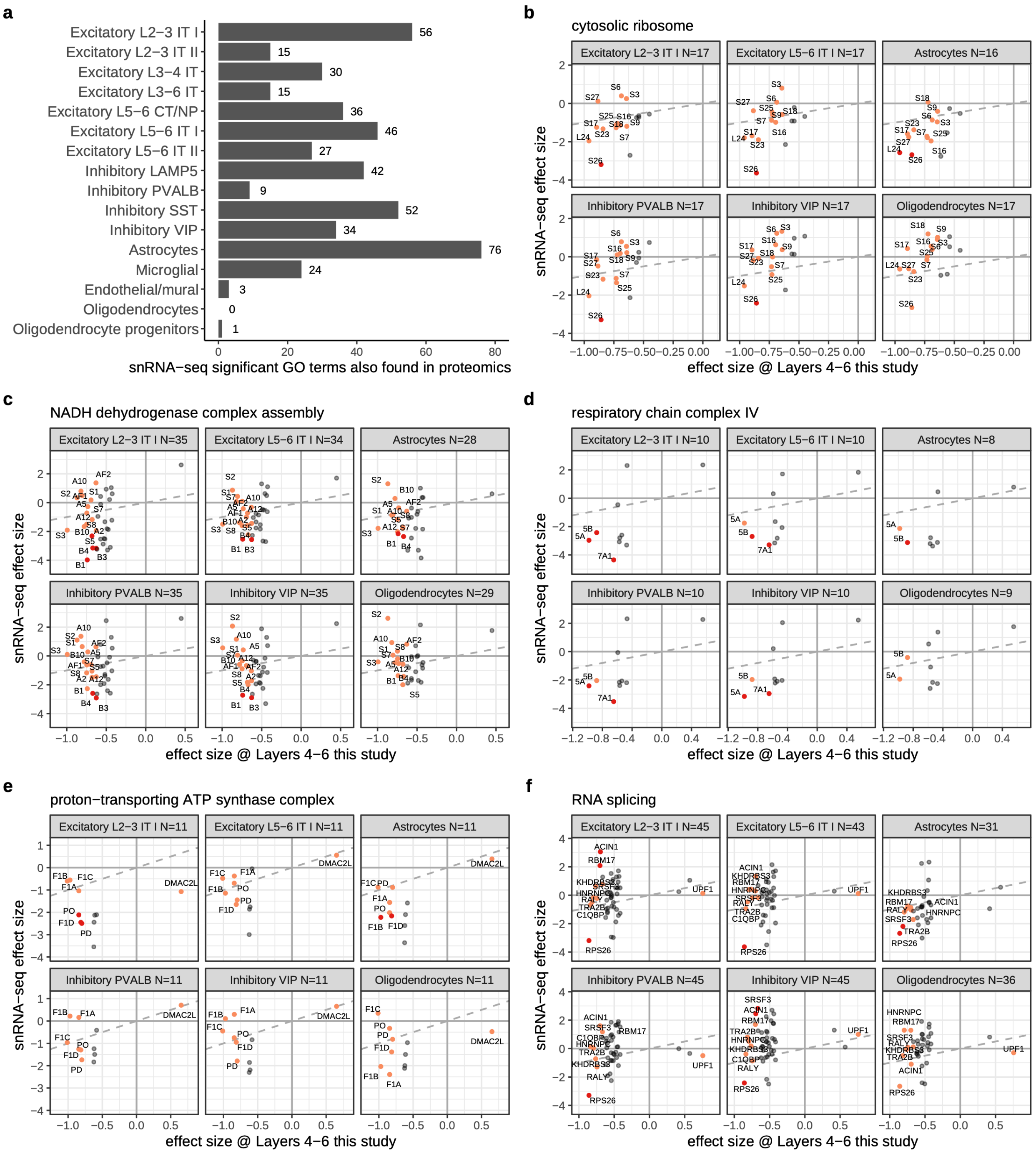
a
Excitatory L2−3 IT I 56
Excitatory L2−3 IT II 15
Excitatory L3−4 IT 30
Excitatory L3−6 IT 15
Excitatory L5−6 CT/NP 36
Excitatory L5−6 IT I 46
Excitatory L5−6 IT II 27
Inhibitory LAMP5 42
Inhibitory PVALB 9
Inhibitory SST 52
Inhibitory VIP 34
Astrocytes 76
Microglial 24
Endothelial/mural 3
Oligodendrocytes 0
Oligodendrocyte progenitors 1
snRNA−seq significant GO terms also found in proteomics
b
cytosolic ribosome
Excitatory L2−3 IT I N=17
Excitatory L5−6 IT I N=17
Astrocytes N=16
Inhibitory PVALB N=17
Inhibitory VIP N=17
Oligodendrocytes N=17
snRNA−seq effect size
effect size @ Layers 4−6 this study
c
NADH dehydrogenase complex assembly
Excitatory L2−3 IT I N=35
Excitatory L5−6 IT I N=34
Astrocytes N=28
Inhibitory PVALB N=35
Inhibitory VIP N=35
Oligodendrocytes N=29
snRNA−seq effect size
effect size @ Layers 4−6 this study
d
respiratory chain complex IV
Excitatory L2−3 IT I N=10
Excitatory L5−6 IT I N=10
Astrocytes N=8
Inhibitory PVALB N=10
Inhibitory VIP N=10
Oligodendrocytes N=9
snRNA−seq effect size
effect size @ Layers 4−6 this study
e
proton−transporting ATP synthase complex
Excitatory L2−3 IT I N=11
Excitatory L5−6 IT I N=11
Astrocytes N=11
Inhibitory PVALB N=11
Inhibitory VIP N=11
Oligodendrocytes N=11
snRNA−seq effect size
effect size @ Layers 4−6 this study
f
RNA splicing
Excitatory L2−3 IT I N=45
Excitatory L5−6 IT I N=43
Astrocytes N=31
Inhibitory PVALB N=45
Inhibitory VIP N=45
Oligodendrocytes N=36
snRNA−seq effect size
effect size @ Layers 4−6 this study

Figure S8. Comparison of schizophrenia (SCZ) associated proteins and pathways between proteomics and snRNA-seq. Variant of main Figure 4, using data from Layers 4-6 for panels b-f.

Comparison between proteomics and snRNA-seq data analogous to main Figure 4, with same layout of panels, but here using proteomics data from Layers 4-6 for panels b-f (whereas main Figure 4 shows data from Layers 1-3).

a) For 338 significant GO terms identified across Layer sets in the proteomics dataset (Fig. 3), the overlap with significant GO terms identified in respective cell-types in the accompanying snRNA-seq study was counted. **b-f**) Comparison between protein effect sizes observed in the proteomics control vs SCZ analysis of Layers 1-3 presented in Fig. 1b (Supplementary Data 2) on the x-axis and respective DESeq2 effect sizes observed in the accompanying snRNA-seq study by Bast et al. on the y-axis for 5 selected gene sets (plot title). The number of proteins in each plot is indicated by 'N' in the title. Gene symbols were shortened for visual clarity as follows, **b**; 'RPS' and 'RPL' were replaced by 'S' and 'L', **c**; 'NDUF' was removed from labels, **d**; 'COX' was removed from labels, **e**; 'APT5' was removed from labels.

Panels indicate 6 representative cell-types. Proteins significant in proteomics (FDR adjusted p-value < 0.05) are shown in red if their respective snRNA-seq FDR adjusted p-value < 0.3 and orange otherwise. Proteins not significant in proteomics are shown in grey. Source data are provided as a Source Data file.

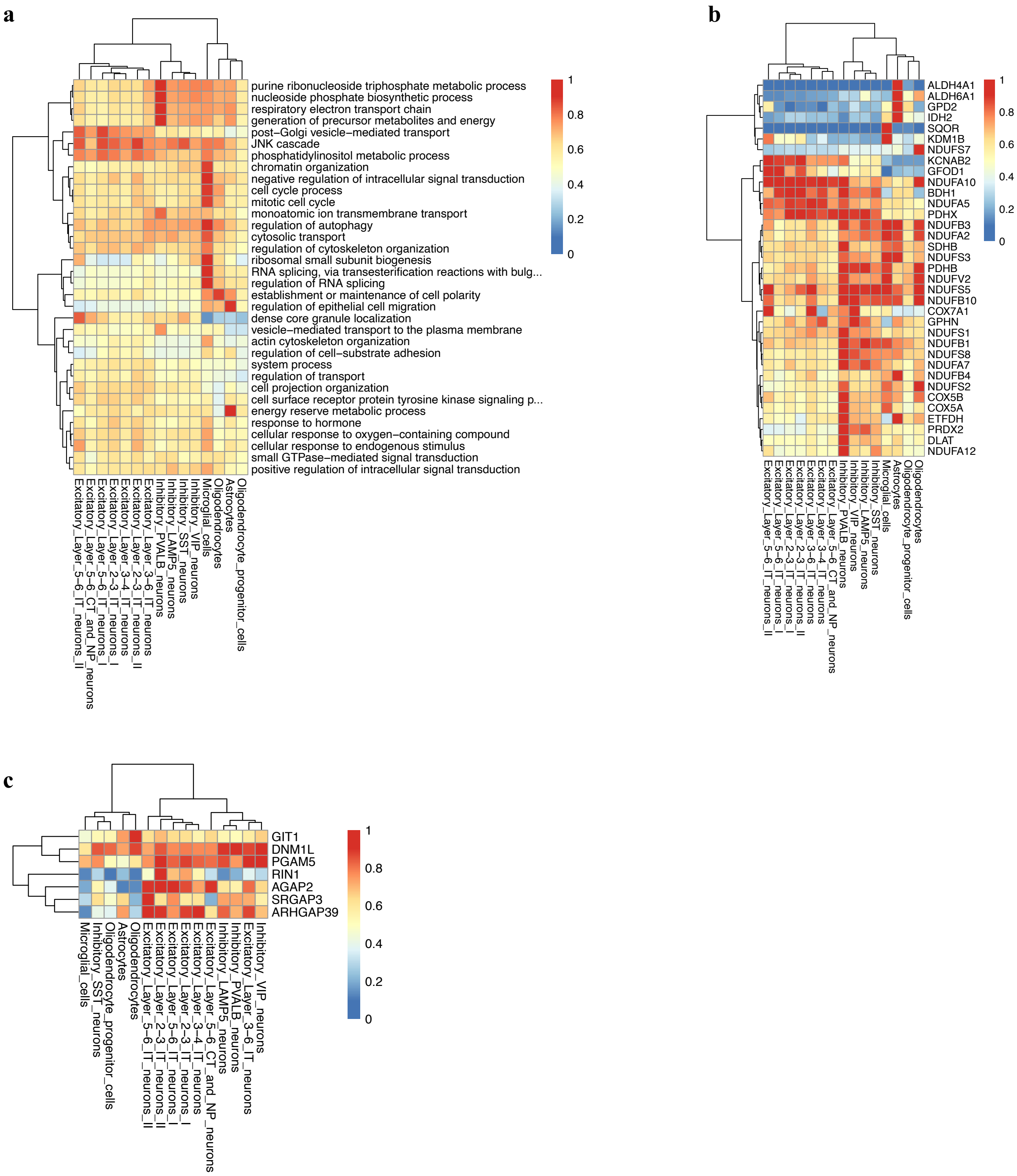
a
purine ribonucleoside triphosphate metabolic process
nucleoside phosphate biosynthetic process
respiratory electron transport chain
generation of precursor metabolites and energy
post–Golgi vesicle–mediated transport
JNK cascade
phosphatidylinositol metabolic process
chromatin organization
negative regulation of intracellular signal transduction
cell cycle process
mitotic cell cycle
monoatomic ion transmembrane transport
regulation of autophagy
cytosolic transport
regulation of cytoskeleton organization
ribosomal small subunit biogenesis
RNA splicing, via transesterification reactions with bulg...
regulation of RNA splicing
establishment or maintenance of cell polarity
regulation of epithelial cell migration
dense core granule localization
vesicle–mediated transport to the plasma membrane
actin cytoskeleton organization
regulation of cell–substrate adhesion
system process
regulation of transport
cell projection organization
cell surface receptor protein tyrosine kinase signaling p...
energy reserve metabolic process
response to hormone
cellular response to oxygen–containing compound
cellular response to endogenous stimulus
small GTPase–mediated signal transduction
positive regulation of intracellular signal transduction
Excitatory_Layer_5–6_IT_neurons_II
Excitatory_Layer_5–6_CT_and_NP_neurons
Excitatory_Layer_5–6_IT_neurons_I
Excitatory_Layer_2–3_IT_neurons_I
Excitatory_Layer_3–4_IT_neurons
Excitatory_Layer_2–3_IT_neurons_II
Excitatory_Layer_3–6_IT_neurons
Inhibitory_PVALB_neurons
Inhibitory_LAMP5_neurons
Inhibitory_SST_neurons
Inhibitory_VIP_neurons
Microglial_cells
Oligodendrocytes
Astrocytes
Oligodendrocyte_progenitor_cells
1
0.8
0.6
0.4
0.2
0
b
ALDH4A1
ALDH6A1
GPD2
IDH2
SQOR
KDM1B
NDUFS7
KCNAB2
GFOD1
NDUFA10
BDH1
NDUFA5
PDHX
NDUFB3
NDUFA2
SDHB
NDUFS3
PDHB
NDUFV2
NDUFS5
NDUFB10
COX7A1
GPHN
NDUFS1
NDUFB1
NDUFS8
NDUFA7
NDUFB4
NDUFS2
COX5B
COX5A
ETFDH
PRDX2
DLAT
NDUFA12
Excitatory_Layer_5–6_IT_neurons_II
Excitatory_Layer_5–6_IT_neurons_I
Excitatory_Layer_2–3_IT_neurons_I
Excitatory_Layer_2–3_IT_neurons_II
Excitatory_Layer_3–6_IT_neurons
Excitatory_Layer_3–4_IT_neurons
Excitatory_Layer_5–6_CT_and_NP_neurons
Inhibitory_PVALB_neurons
Inhibitory_VIP_neurons
Inhibitory_LAMP5_neurons
Inhibitory_SST_neurons
Microglial_cells
Astrocytes
Oligodendrocyte_progenitor_cells
Oligodendrocytes
1
0.8
0.6
0.4
0.2
0
c
GIT1
DNM1L
PGAM5
RIN1
AGAP2
SRGAP3
ARHGAP39
Microglial_cells
Inhibitory_SST_neurons
Oligodendrocyte_progenitor_cells
Astrocytes
Oligodendrocytes
Excitatory_Layer_5–6_IT_neurons_II
Excitatory_Layer_2–3_IT_neurons_II
Excitatory_Layer_5–6_IT_neurons_I
Excitatory_Layer_2–3_IT_neurons_I
Excitatory_Layer_3–4_IT_neurons
Excitatory_Layer_5–6_CT_and_NP_neurons
Inhibitory_LAMP5_neurons
Inhibitory_PVALB_neurons
Excitatory_Layer_3–6_IT_neurons
Inhibitory_VIP_neurons
1
0.8
0.6
0.4
0.2
0

Figure S9. Cell type expression patterns of genes across GO terms enriched in proteomics.

Gene specificity scores were calculated by rescaling gene TPM values from the Bast et al. snRNA-seq dataset by their respective maximum value within each cell type. GO term specificity scores were computed as the (20%) trimmed mean of gene specificity scores from significant genes (in proteomics control vs SCZ, Fig 1b-c, Supplementary Data 2) per GO term. **a**) Significant GO BP terms from control versus SCZ analyses across both Layer sets in the proteomics data (Supplementary Data 5) were reduced to a non-redundant set of GO terms using GOAT (default options) and subselected for those with 3 or more significant genes in the proteomics data (Supplementary Data 2). The resulting 34 GO terms are shown on the y-axis, cell types are shown on x-axis and colors represent the respective GO term specificity scores (based on snRNA-seq expression profiles of gene constituents). **b-c**) To illustrate heterogeneity of expression patterns across cell types at the gene-level, heatmaps of GO terms 'oxidoreductase activity' (**b**) and 'GTPase activator activity' (**c**) are colored by gene specificity scores. The y-axis shows the subset of gene constituents per GO term that were significant in the proteomic analyses of control versus SCZ in either Layer set (Supplementary Data 2). Source data are provided as a Source Data file.

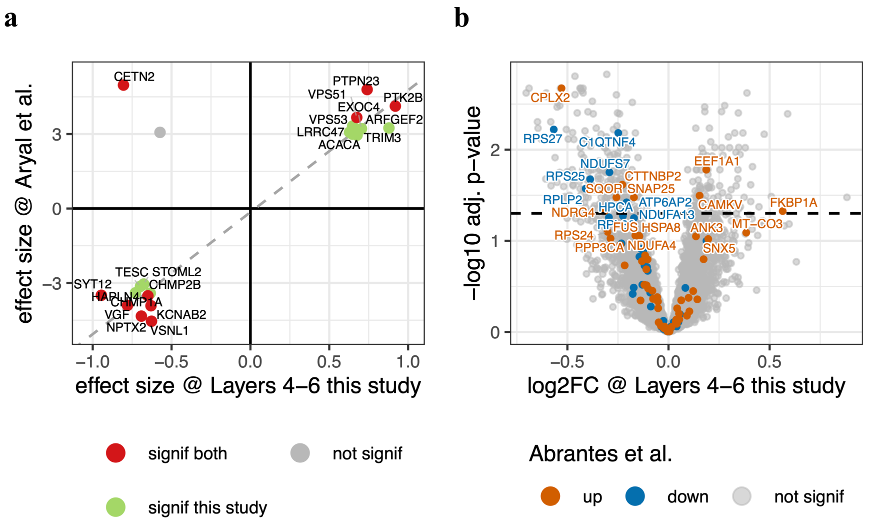

Figure S10. Comparative analysis of data in this study with previously published schizophrenia (SCZ) datasets. Variant of main Figure 5c-d, using data from Layers 4-6.

Panels **a** and **b** are analogous to main Figure 5c-d, respectively, but here using proteomics data from Layers 4-6 (whereas main Figure 5c-d shows data from Layers 1-3).

a) A high degree of similarity in dysregulation is observed for 48 proteins differentially expressed at FDR adjusted p-value < 0.1 in both our study and Aryal et al. The x-axis shows SCZ effect sizes from Layers 4-6 (Fig. 1c). The y-axis shows effect sizes reported by Aryal et al. FDR adjusted p-value < 0.05 was used to classify proteins as significant; red: significant in both studies, green: significant in either study, grey: not significant in either. The dashed line shows a trendline fitted by robust linear regression. **b**) Control vs SCZ analysis of Layers 4-6 presented in Fig. 1c, with proteins color-coded by their regulation after Olanzapine administration in mice as observed with RNA sequencing by Abrantes et al. Up/down-regulated proteins (FDR adjusted p-value < 0.05 in Abrantes et al.) are depicted in orange/blue. Note the anti-correlation of some proteins (e.g. synaptic proteins CPLX2, SNAP25) observed with lower abundance in SCZ patients in this study (x-axis) and the opposite effect observed for Olanzapine treatment in mice by Abrantes et al (color). Source data are provided as a Source Data file.

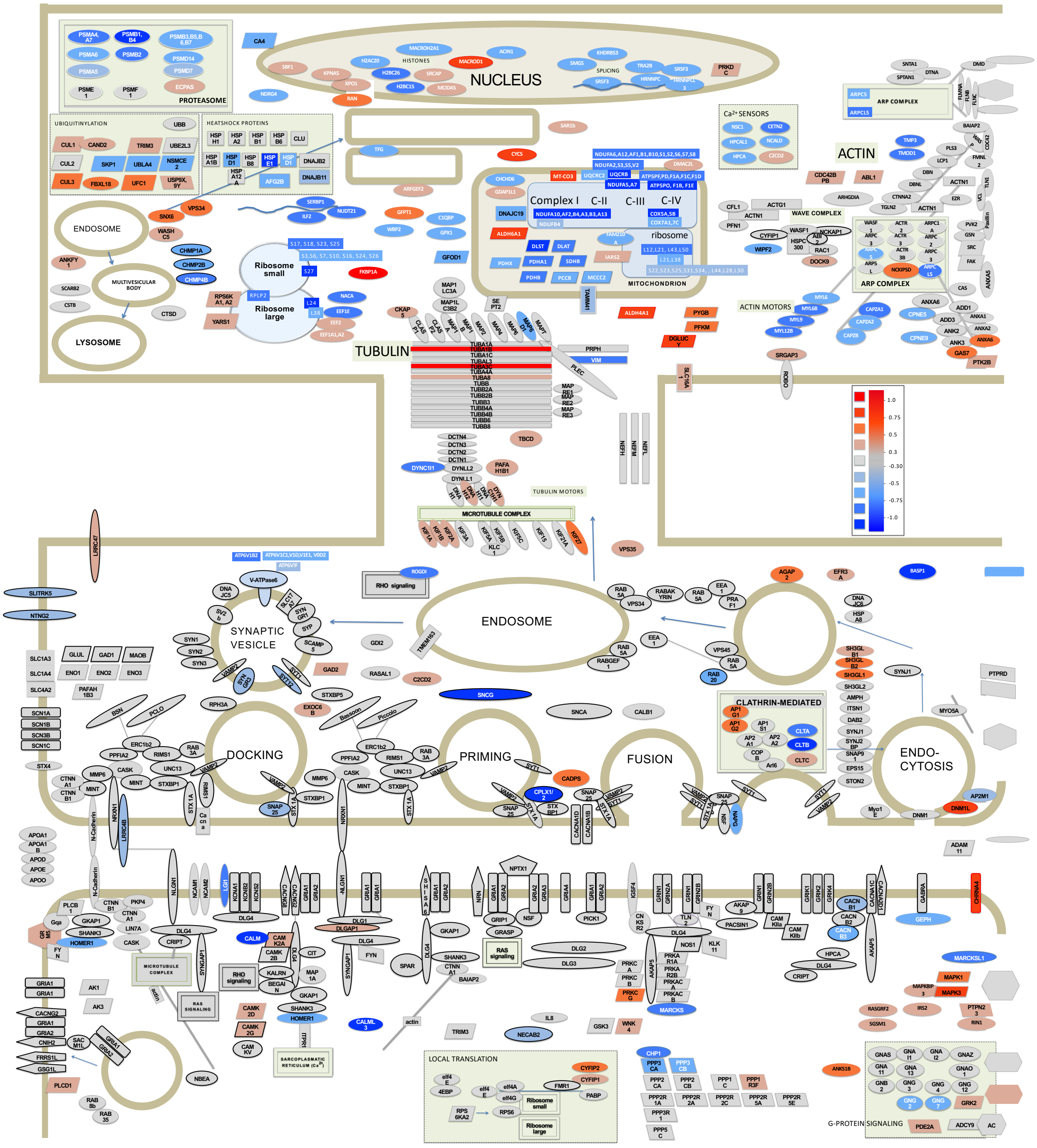

PROTEASOME
PSMA4, A7
PSMB1, B4
PSMB3,B5,B6,B7
PSMA6
PSMB2
PSMD14
PSMA5
PSMD7
ECPAS
PSME 1
PSMF 1
CA4
NUCLEUS
HISTONES
SPLICING
MACROH2A1
ACIN1
KHDRBS3
H2AC20
H2BC26
H2BC15
MACROD1
SBF1
KPNA5
XPO1
RAN
SRCAP
MCIDAS
SMG5
TRA2B
SRSF3
HNRNPC
SRSF1
HNRNPLL
PRKD C
NDRG4
UBIQUITINYLATION
UBB
CUL1
CAND2
TRIM3
UBE2L3
CUL2
SKP1
UBLA4
NSMCE 2
CUL3
FBXL18
UFC1
USP9X, 9Y
HEATSHOCK PROTEINS
HSP H1
HSP A2
HSP B1
HSP B6
CLU
HSP A1B
HSP D1
HSP B8
HSP E1
HSP D1
DNAJB2
HSP A12 A
AFG3L2
DNAJB11
TFG
SAR1b
ARFGEF2
CYCS
MT-CO3
UQCRC2
UQCRB
NDUFA6,A12,AF1,B1,B10,S1,S2,S6,S7,S8
NDUFA2,S3,S5,V2
DMAC2L
ATP5PF,PO,F1A,F1C,F1D
NDUFAS,A7
ATP5PO, F1B, F1E
CHCHD6
GDAP1L1
Complex I
C-II
C-III
C-IV
DNAJC19
NDUFA10,AF2,B4,A3,B3,A13
COX5A,5B
NDUFB4
COX7A1,7C
ALDH6A1
ribosome
DLST
DLAT
FAM210 A
IARS2
PDHX
PDHA1
SDHB
L12,L21, L43,L50
L21,L38
PDHB
PCCB
MCCC2
S22,S23,S25,S31,S34, , L44,L28,L30
MITOCHONDRION
ENDOSOME
SNX6
VPS34
WASH C5
ANKFY 1
CHMP1A
CHMP2B
CHMP4B
MULTIVESICULAR BODY
SCARB2
CSTB
CTSD
LYSOSOME
SERBP1
NUDT21
ILF2
GFPT1
C1QBP
WBP2
GPX1
S17, S18, S23, S25
S3,S6, S7, S10, S16, S24, S26
S27
Ribosome small
FKBP1A
RPS6K A1, A2
RPLP2
YARS1
L24
L38
NACA
EEF1E
EEF2
EEF1A1,A2
Ribosome large
GFOD1
MAP1 LC3A
MAP1L C3B2
SE PT2
CKAP 5
TUBULIN
TUBA1A
TUBA1B
TUBA1C
TUBAL3
TUBA3C
TUBA4A
TUBA8
TUBB
TUBB2A
TUBB2B
TUBB3
TUBB4A
TUBB4B
TUBB6
TUBB8
PRPH
VIM
PLEC
MAP RE1
MAP RE2
MAP RE3
TANMM
ALDH4A1
PYGB
PFKM
DGLUC Y
SLC16A 1
DCTN4
DCTN3
DCTN2
DCTN1
DYNLL2
DYNLL1
DYNC1I1
PAFA H1B1
TBCD
TUBULIN MOTORS
MICROTUBULE COMPLEX
KIF1A
KIF1B
KIF3A
KIF5A
KIF5B
KLC 1
KIF5C
KIF15
KIF21A
KIF27
NEFH
NEFM
NEFL
Ca²⁺ SENSORS
NSC1
CETN2
HPCAL1
NCALD
HPCA
C2CD2
ACTIN
ARP COMPLEX
ARPC5
ARPCL5
SNTA1
DTNA
SPTAN1
DMD
BAIAP2
TMP3
TMOD1
PLS3
CDC42B PB
ABL1
DBN
ACTN1
ARHGDIA
CTNNA2
TGLN2
ACTN1
EZR
CFL1
ACTG1
ACTN1
PFN1
WAVE COMPLEX
CYFIP1
WASF1
NCKAP1
ABI 2
HSPC 300
WIPF2
RAC1
DOCK9
NCKIPSD
ARP COMPLEX
PYK2
GSN
SRC
FAK
CAS
ACTIN MOTORS
MYL6
MYL6B
MYL9
MYL12B
CAPZA1
CAPZA2
CAPZB
CPNE5
CPNE9
ANXA6
ADD1
ADD3
ANXA1
ANK2
ANXA2
ANK3
ANXA6
GAS7
PTK2B
SRGAP3
ROBO
1.0
0.75
0.5
0.3
-0.30
-0.5
-0.75
-1.0
LRRC47
VPS35
ATP6V1B2
ATP6V1C1,V1D,V1E1, V0D2
ATP6V1F
V-ATPase6
ROGDI
RHO signaling
SLITRK5
NTNG2
DNA JC5
SV2 b
SYN GR1
SYP
SYNAPTIC VESICLE
SCAMP 5
SYN1
SYN2
SYN3
VAMP2
SYN GR3
SYT1
SYT1V
GDI2
TMEM163
ENDOSOME
RAB 5A
VPS34
RABAK YRIN
RAB 5A
EEA 1
PRA F1
AGAP 2
EFR3 A
BASP1
DNA JC6
HSP A8
EEA 1
RAB 5A
RABGEF 1
VPS45
RAB 5A
RAB 20
SLC1A3
SLC1A4
SLC4A2
GLUL
GAD1
MAOB
ENO1
ENO2
ENO3
PAFAH 1B3
GAD2
RASAL1
C2CD2
SNCG
STXBP5
EXOC6 B
RPH3A
SNCA
CALB1
SH3GL B1
SH3GL B2
SH3GL1
SH3GL2
AMPH
ITSN1
DAB2
SYNJ1
SYNJ2 BP
SNAP9 1
EPS15
STON2
SYNJ1
CLATHRIN-MEDIATED
AP1 G1
AP1 G2
AP1 S1
CLTA
CLTB
AP2 A1
AP2 A2
COP B
Arl6
CLTC
PTPRD
MYO5A
ENDO-CYTOSIS
AP2M1
DNM1L
DNM1
Myo1 E
SCN1A
SCN1B
SCN3B
SCN1C
STX4
BSN
PCLO
ERC1b2
PPFIA2
RIMS1
RAB 3A
DOCKING
UNC13
CASK
MMP6
MINT
STXBP1
CTNN A1
CTNN B1
Bassoon
Piccolo
PRIMING
CADPS
CPLX1/ 2
FUSION
SNAP 25
STX BP1
CACNA1D
CACNA1B
NSF
NAPG
APOA1
APOA1 B
APOD
APOE
APOO
N-Cadherin
NRXN1
LRRC4B
NLGN1
NCAM1
NCAM2
KCNA1
KCNB2
KCNS2
CACNG8
GRIA2
DLG4
CAM K2A
CALM
DLGAP1
DLG1
GKAP1
SHANK3
CTNN A1
BAIAP2
NPTX1
NRN
GRIA1
GRIP1
GRASP
NSF
RAS signaling
PICK1
DLG2
DLG3
GRIN1
GRIN2A
GRIN2B
AKAP 9
PACSIN1
CAM KIIa
CAM KIIb
KIF21A
GRIK1
GRIK2
GRIK4
CACN B1
CACN B2
CACN B3
CACNA1C
HPCA
CRIPT
AKAP5
GABRA
GEPH
CHRNA4
MARCKSL1
MAPK1
MAPK8IP 3
MAPK3
RASGRF2
IRS2
PTPN2 3
SGSM1
RIN1
PLCB 1
Gqa
PKP4
CTNN B1
CTNN A1
LIN7A
CASK
CRIPT
HOMER1
FY N
GRIA1
GRIA1
AK1
AK3
CACNG2
GRIA1
GRIA2
CNIH2
FRRS1L
GSG1L
SAC M1L
PLCD1
RAB 8b
RAB 35
MICROTUBULE COMPLEX
RHO signaling
RAS SIGNALING
SYNGAP1
KALRN
BEGAI N
CAMK 2B
CAMK 2D
CAMK 2G
CAM KV
MAP 1A
CIT
FYN
SPAR
SARCOPLASMATIC RETICULUM (Ca²⁺)
ITPR1
NBEA
CALML 3
actin
TRIM3
IL8
NECAB2
GSK3
WNK 4
NOS1
KLK 11
PRKC A
PRKC B
PRKC G
PRKA R1A
PRKA R2B
PRKAC A
PRKAC B
MARCKS
CNKS R2
TLN 2
LOCAL TRANSLATION
eIf4 E
4EBP
eIf4 E
eIf4A
eIf4G
FMR1
CYFIP2
CYFIP1
PABP
Ribosome small
Ribosome large
RPS 6KA2
RPS6
CHP1
PPP3 CA
PPP3 CB
PPP2 CA
PPP2 CB
PPP2R 1A
PPP2R 2A
PPP2R 2C
PPP1 C
PPP1 R3F
PPP2R 5A
PPP2R 5E
PPP3R 1
PPP5 C
ANKS1B
GNAS
GNA I1
GNA I2
GNAZ
GNA 11
GNA 13
GNAO 1
GNB 2
GNG 3
GNG 4
GNG 12
GNG 2
GNG 7
GRK2
G-PROTEIN SIGNALING
PDE2A
ADCY9
AC
ADAM 11

Figure S11. Schematic representation of synaptic subcellular localizations for proteins differentially regulated in schizophrenia.

Differentially regulated proteins in schizophrenia are depicted at their canonical localization. Higher (red) and lower (blue) expression in schizophrenia as indicated by color coding of foldchanges (see legend in middle part of the figure). For the log2 foldchanges shown in this figure, the same limma regression model with DEqMS post-processing was used as with analyses shown in Figure 1b-c, but here using all samples in the dataset and estimating the effect of SCZ across both Layer sets (using a block design in limma, see Methods). Note that most of the dysregulated proteins are not specifically synaptic as becomes evident from a tissue homogenate proteomics approach. Source data are provided as a Source Data file.
