# Supplementary material for: Human brain prefrontal cortex proteomics identifies compromised energy metabolism and neuronal function in Schizophrenia
Source: Nat Commun. 2026 Jan 29;17:2131. doi: 10.1038/s41467-026-68950-y (PMC12957516; doi:10.1038/s41467-026-68950-y)
Supplement: Supplementary file 2 — Description of Additional Supplementary Files [file 41467_2026_68950_MOESM2_ESM.pdf]

Description of Additional Supplementary Files

File name: Supplementary Data 1
Description: Sample metadata for all proteomic samples.

File name: Supplementary Data 2
Description: Statistical analyses using limma linear regression modeling, postprocessed with DEqMS (Methods), for independent control-vs-SCZ analyses per Layer set from Figures 1b-d and between-Layers comparison from Figure 2a.

File name: Supplementary Data 3
Description: pQTL significant hits at FDR adjusted p-value < 0.05 from Figure 2b.

File name: Supplementary Data 4
Description: Stratified LDSC analyses from Figure 2c.

File name: Supplementary Data 5
Description: GO analyses using GOAT from Figure 3.

File name: Supplementary Data 6
Description: SynGO analysis via www.syngoportal.org from Figure 3f.
