# Supplementary material for: Human brain prefrontal cortex proteomics identifies compromised energy metabolism and neuronal function in Schizophrenia
Source: Nat Commun. 2026 Jan 29;17:2131. doi: 10.1038/s41467-026-68950-y (PMC12957516; doi:10.1038/s41467-026-68950-y)
Supplement: Supplementary file 9 — Reporting Summary [file 41467_2026_68950_MOESM9_ESM.pdf]

nature portfolio

Corresponding author(s): August B. Smit

Last updated by author(s): Dec 30, 2025

Reporting Summary

Nature Portfolio wishes to improve the reproducibility of the work that we publish. This form provides structure for consistency and transparency in reporting. For further information on Nature Portfolio policies, see our Editorial Policies and the Editorial Policy Checklist.

Statistics

For all statistical analyses, confirm that the following items are present in the figure legend, table legend, main text, or Methods section.

n/a	Confirmed	
☐	☒	The exact sample size (*n*) for each experimental group/condition, given as a discrete number and unit of measurement
☐	☒	A statement on whether measurements were taken from distinct samples or whether the same sample was measured repeatedly
☐	☒	The statistical test(s) used AND whether they are one- or two-sided<br>*Only common tests should be described solely by name; describe more complex techniques in the Methods section.*
☐	☒	A description of all covariates tested
☐	☒	A description of any assumptions or corrections, such as tests of normality and adjustment for multiple comparisons
☐	☒	A full description of the statistical parameters including central tendency (e.g. means) or other basic estimates (e.g. regression coefficient) AND variation (e.g. standard deviation) or associated estimates of uncertainty (e.g. confidence intervals)
☐	☒	For null hypothesis testing, the test statistic (e.g. *F*, *t*, *r*) with confidence intervals, effect sizes, degrees of freedom and *P* value noted<br>*Give P values as exact values whenever suitable.*
☒	☐	For Bayesian analysis, information on the choice of priors and Markov chain Monte Carlo settings
☐	☒	For hierarchical and complex designs, identification of the appropriate level for tests and full reporting of outcomes
☐	☒	Estimates of effect sizes (e.g. Cohen's *d*, Pearson's *r*), indicating how they were calculated

Our web collection on statistics for biologists contains articles on many of the points above.

Software and code

Policy information about availability of computer code

Data collection	not applicable; collected data are mass-spectrometric measurements of brain material from Human donors
Data analysis	As detailed in the Methods section of the manuscript;<br>Raw mass-spectrometry data was analyzed using DIA-NN 1.8, then processed with MS-DAP 1.0.4<br>Release 2021-04 of the UniProt human proteome (SwissProt and TrEMBL, canonical and additional isoforms) was used in DIA-NN analyses<br>Statistical analyses were performed using limma 3.52.2 and DEqMS 1.14.0 (using the integration in MS-DAP)<br>GO analyses were performed using GOAT 1.1.2<br>Release 2025-01-01 of the NCBI gene2go database was used for GO analyses.<br>SynGO analyses were performed using the www.syngoportal.org webtools (database release 1.2)<br>pQTL analyses were performed using RICOPILI (2019) and QTLtools 1.3.1

For manuscripts utilizing custom algorithms or software that are central to the research but not yet described in published literature, software must be made available to editors and reviewers. We strongly encourage code deposition in a community repository (e.g. GitHub). See the Nature Portfolio guidelines for submitting code & software for further information.

Data

Policy information about availability of data

All manuscripts must include a data availability statement. This statement should provide the following information, where applicable:

- Accession codes, unique identifiers, or web links for publicly available datasets
- A description of any restrictions on data availability
- For clinical datasets or third party data, please ensure that the statement adheres to our policy

The mass spectrometry proteomics data have been deposited in the ProteomeXchange Consortium via the PRIDE partner repository with the dataset identifier PXD058441. Source data are provided with this paper.

Research involving human participants, their data, or biological material

Policy information about studies with human participants or human data. See also policy information about sex, gender (identity/presentation), and sexual orientation and race, ethnicity and racism.

Reporting on sex and gender	All donor tissue is described in Table S1, which includes the sex of anonymised individuals. The dataset contains a mix of males and females in both control and schizophrenia cases. Due to a lack of statistical power we did not further investigate differences between male and female in context of schizophrenia (i.e. a much larger cohort would be needed).
Reporting on race, ethnicity, or other socially relevant groupings	Not applicable; brain donors were grouped by their clinical diagnosis
Population characteristics	All donor tissue is described in Table S1. We used linear regression models to compare between clinical diagnoses (control vs schizophrenia) and used the tissue source (/origin, a technical covariate), gel (technical covariate), post mortem delay (technical covariate) and age (biological covariate) as covariates. Exploratory data analyses for covariates are available in Supporting Information.
Recruitment	Post-mortem donor tissue was obtained from 3 collections:<br>- The Netherlands Brain Bank<br>- Andrew Dwork<br>- Craig Stockmeier
Ethics oversight	Post-mortem brain tissue of the dorsal prefrontal cortex (DPFC) of 47 clinically assessed schizophrenia cases and 49 healthy controls were obtained from three sites. Cases and controls were selected based on clinical and neuropathological reports (evaluated by PFS). We obtained 3 controls and 8 cases from The Netherlands Brain Bank (NBB, denoted as BA in sample metadata). Provision of samples and study design was approved by the NBB's Tissue Advisory Board. We obtained 19 controls and 13 cases from the brain sample collection of Dr Andrew Dwork (denoted as AA in sample metadata). Provision of samples from the Macedonian/New York State Psychiatric Institute Brain Collection and study design was approved by the New York State Psychiatric Institute Institutional Review Board. We obtained 27 controls and 29 cases from the brain sample collection of Craig Stockmeier (denoted as CB in sample metadata). Provision of samples and study design was approved by the IRB of the University of Mississippi Medical Center (Protocol 1999-1002) and the University Hospitals Case Medical Center (Protocol 11-88-233). All brain tissue was collected from donors with written informed consent for brain autopsy and the use of brain tissue and clinical information for research purposes, complying with all relevant regulations at each site of sample collection.

Note that full information on the approval of the study protocol must also be provided in the manuscript.

Field-specific reporting

Please select the one below that is the best fit for your research. If you are not sure, read the appropriate sections before making your selection.

☒ Life sciences ☐ Behavioural & social sciences ☐ Ecological, evolutionary & environmental sciences

For a reference copy of the document with all sections, see nature.com/documents/nr-reporting-summary-flat.pdf

Life sciences study design

All studies must disclose on these points even when the disclosure is negative.

Sample size	For neuropsychiatric disorders the changes in the levels of proteins are expected to be minimal. This argues for an approach which includes as many samples as possible into such a study. However, availability of patient material is a challenge. Through collaborations between 3 cohorts of sample collection, we were able to collect post-mortem tissue from 47 SCZ cases (and 49 sex and age matched healthy controls). This is a unique cohort of patient material with the same sample size as the currently largest SCZ proteomics study (MacDonald et al. 2020 - 48 SCZ cases and 48 controls).
Data exclusions	No data were excluded from analysis
Replication	The study features mass-spectrometric measurement of post-mortem brain tissue from 96 donors. It is not practically feasible to collect a second cohort of this size to replicate the study. However, we did collect 2 different tissues from each donor (cortical layers 1-3 and layers 4-6) and while our intent was to study differential regulation of schizophrenia-affected proteins between these layers we found that the

	control-vs-schizophrenia effect was highly correlated between both layers. Ergo, this part of our study is at least a technical replication of the sample collection, processing and mass-spectrometric assessment of these tissues; schizophrenia-associated proteins that we found in Layers 1-3 were corroborated by highly correlated measurements of Layers 4-6. Note that each tissue sample was independently processed in the lab and independently subjected to mass-spectrometry.
Randomization	The tissue dissection was performed by Anke Dijkstra. She was not aware of the disease status of the LCM-collected tissue. Collected samples were subsequently allocated to experimental groups according to clinical diagnosis and cortical layer; these groupings were then used to perform block randomization of all samples during sample processing and mass-spectrometry to avoid introducing confounding effects.
Blinding	The tissue dissection was performed by Anke Dijkstra. She was not aware of the disease status of the LCM-collected tissue.

Reporting for specific materials, systems and methods

We require information from authors about some types of materials, experimental systems and methods used in many studies. Here, indicate whether each material, system or method listed is relevant to your study. If you are not sure if a list item applies to your research, read the appropriate section before selecting a response.

Materials & experimental systems

n/a	Involved in the study
☐	☒ Antibodies
☒	☐ Eukaryotic cell lines
☒	☐ Palaeontology and archaeology
☒	☐ Animals and other organisms
☒	☐ Clinical data
☒	☐ Dual use research of concern
☒	☐ Plants

Methods

n/a	Involved in the study
☒	☐ ChIP-seq
☒	☐ Flow cytometry
☒	☐ MRI-based neuroimaging

Antibodies

Antibodies used	IC16 (amyloid beta), diluted 1:800, a kind gift of Prof Dr Korth, Heinrich Heine University, Düsseldorf, Germany<br>AT8 (tau), dilution 1:800, Pierce Biotechnology, Rockford, IL<br>p62 (clone 3/P62 LCK LIGAND), dilution 1:1000, BD Transduction Laboratories, San Jose, CA, USA<br>SMI-32, dilution 1:8000, Biolegend, San Diego, CA, USA
Validation	*Describe the validation of each primary antibody for the species and application, noting any validation statements on the manufacturer's website, relevant citations, antibody profiles in online databases, or data provided in the manuscript.*

Plants

Seed stocks	*Report on the source of all seed stocks or other plant material used. If applicable, state the seed stock centre and catalogue number. If plant specimens were collected from the field, describe the collection location, date and sampling procedures.*
Novel plant genotypes	*Describe the methods by which all novel plant genotypes were produced. This includes those generated by transgenic approaches, gene editing, chemical/radiation-based mutagenesis and hybridization. For transgenic lines, describe the transformation method, the number of independent lines analyzed and the generation upon which experiments were performed. For gene-edited lines, describe the editor used, the endogenous sequence targeted for editing, the targeting guide RNA sequence (if applicable) and how the editor was applied.*
Authentication	*Describe any authentication procedures for each seed stock used or novel genotype generated. Describe any experiments used to assess the effect of a mutation and, where applicable, how potential secondary effects (e.g. second site T-DNA insertions, mosiacism, off-target gene editing) were examined.*
